# Asthma prediction via affinity graph enhanced classifier: a machine learning approach based on routine blood biomarkers

**DOI:** 10.1186/s12967-024-04866-9

**Published:** 2024-01-24

**Authors:** Dejing Li, Stanley Ebhohimhen Abhadiomhen, Dongmei Zhou, Xiang-Jun Shen, Lei Shi, Yubao Cui

**Affiliations:** 1 Present Address: Department of Respiratory, The Affiliated Wuxi People’s Hospital of Nanjing Medical University, Wuxi, 214023 China; 2 School of Computer Science and Communication Engineering, JiangSu University, Zhenjiang, JiangSu 212013 China; 3 Department of Computer Science, University of Nigeria, Nsukka, Nigeria; 4 Clinical Research Center, The Affiliated Wuxi People’s Hospital of Nanjing Medical University, Wuxi, 214023 China; 5 Department of Clinical Laboratory, Shuguang Hospital Affiliated to Shanghai University of Chinese Traditional Medicine, Shanghai, 201203 China

**Keywords:** Asthma prediction, Asthma, Affinity graph, Feature selection

## Abstract

**Background:**

Asthma is a chronic respiratory disease affecting millions of people worldwide, but early detection can be challenging due to the time-consuming nature of the traditional technique. Machine learning has shown great potential in the prompt prediction of asthma. However, because of the inherent complexity of asthma-related patterns, current models often fail to capture the correlation between data samples, limiting their accuracy. Our objective was to use our novel model to address the above problem via an Affinity Graph Enhanced Classifier (AGEC) to improve predictive accuracy.

**Methods:**

The clinical dataset used in this study consisted of 152 samples, where 24 routine blood markers were extracted as features to participate in the classification due to their ease of sourcing and relevance to asthma. Specifically, our model begins by constructing a projection matrix to reduce the dimensionality of the feature space while preserving the most discriminative features. Simultaneously, an affinity graph is learned through the resulting subspace to capture the internal relationship between samples better. Leveraging domain knowledge from the affinity graph, a new classifier (AGEC) is introduced for asthma prediction. AGEC’s performance was compared with five state-of-the-art predictive models.

**Results:**

Experimental findings reveal the superior predictive capabilities of AGEC in asthma prediction. AGEC achieved an accuracy of 72.50%, surpassing FWAdaBoost (61.02%), MLFE (60.98%), SVR (64.01%), SVM (69.80%) and ERM (68.40%). These results provide evidence that capturing the correlation between samples can enhance the accuracy of asthma prediction. Moreover, the obtained $p$ values also suggest that the differences between our model and other models are statistically significant, and the effect of our model does not exist by chance.

**Conclusion:**

As observed from the experimental results, advanced statistical machine learning approaches such as AGEC can enable accurate diagnosis of asthma. This finding holds promising implications for improving asthma management.

## Background

Asthma affects 235 million people globally [1], making it one of the most common chronic diseases in the world, according to the World Health Organization [2]. Specifically, asthma is characterized by inflammation of the airways, which results in symptoms such as wheezing, shortness of breath, and chest tightness [3, 4]. In order to avoid exacerbations and hospitalizations, asthma must be accurately and promptly diagnosed for effective management and treatment of the disease [5]. Conventional diagnostic methods often combine medical history, physical examination, and lung function tests. Apart from the fact that these tests are expensive, atypical symptoms in some patients can result in delayed or missed diagnoses. Moreover, asthma in young children can be very difficult to diagnose, and traditional methods may exacerbate the situation due to their time-consuming nature [6].

With the advancement of machine learning (ML), there is a growing interest [7–13] in predicting asthma using computational techniques to analyze medical data, identify patterns and generate predictions that can assist healthcare providers in early and more accurate diagnoses of asthma. Typical predictive models include Decision Trees [14], Random Forests [15], Support Vector Machines (SVMs) [16], Neural Networks [17], and Bayesian Networks [18]. Despite the successes of these classical ML models, they often cannot capture the internal relationships between data samples, making them less robust for complex medical conditions like asthma. This inadequacy could arise from a combination of limitations in model complexity, algorithmic constraints, and insufficient adaptability to dynamic and intricate patterns within the asthma data. Addressing this problem may help unlock the full potential of ML in the prediction and management of asthma. Recently, graph-based learning (GBL) [19, 20] has emerged as a promising method for capturing correlation between data samples. GBL has found widespread use in subspace clustering [21–23] via an affinity graph construction. Here, each sample is reconstructed by a linear combination of other samples in the same subspace. According to Lu et al. [24], such subspace representation can allow for a more detailed understanding of data and can reveal important patterns that might be missed by traditional clustering methods.

Inspired by this, a new ML approach, which uses an affinity graph enhanced classifier (AGEC) for asthma prediction, is proposed in this paper. As far as we know, this is the first study that directly exploits an affinity graph for classification. Accordingly, we demonstrate through experimental evaluation with existing ML models that AGEC can tackle the above problem and improve asthma prediction accuracy. Therefore, we hope that the results of our study can assist the clinical community in the prompt prediction and management of asthma.

## Methods

### Data collection

The datasets used in this study contained 152 records collected from asthma patients in the Affiliated Shuguang Hospital of Shanghai Traditional Chinese Medicine University. Before the study was conducted, ethical approval was obtained from the relevant ethics committee at the Affiliated Shuguang Hospital of Shanghai Traditional Chinese Medicine University. The sample population in the dataset ranges between 20 and 100 years old. Of the 152 samples in the dataset, 18.4% are between 20 and 40 years old, 47.4% are between 50 and 69 years old, and 34.2% are over 70 years old. The age distribution of the sample indicates that the majority of the participants were between 50 and 69 years old. In terms of gender, the dataset includes 40% males and 60% females, with a male to female ratio of roughly 4:6 (see Table 1 for a summary of the dataset). For each record, twenty-four indicators which include complete blood count differentials and red blood cell indices were extracted for use as candidate predictors in the classification procedure, as shown in Table 2. The diagnosis results were used as the label. In this study, there are five possible diagnosis categories: asthma, bronchial asthma, sputum turbidiosis, non-critical-bronchial asthma, and no diagnosis.

**Table 1:** Summary of the characteristics of the dataset

Samples	Features	% of men	% of woman	Age distribution of samples	% of samples with age between 20–40 years	% of samples with age between 50–69 years	% of samples with age above 70 years
152	24	40	60	Between 20–100 years	18.4	47.4	34.2

**Table 2:** Twenty-four clinical indicators extracted as candidate predictors

White blood cell (WBC)	Neutrophil percentage (NE%)	Lymphocyte percentage (LY%)	Monocyte percentage (MO%)	Eosinophil percentage (EO%)	Basophil percentage (BA%)	Neutrophil count—absolute (NE#)	Lymphocyte count—absolute (LY#)
Monocyte count—absolute (MO#	Eosinophil count—absolute (EO#)	Basophil count—absolute (BA#)	Red blood cell (RBC)	Hemoglobin (HGB)	Hematocrit (HCT)	Mean corpuscular volume (MCV)	Mean corpuscular hemoglobin (MCH)
Mean corpuscular hemoglobin concentration (MCHC)	Red cell distribution width (RDW)	Platelet count (PLT)	Platelet distribution width (PDW)	Platelet crit (PCT)	Mean platelet volume (MPV)	C-reactive protein (CRP)	Serum amyloid A (SAA)

### Model formulation

This section describes the formulation of our proposed model. Firstly, in order to transform the raw data into appropriate format that can be used by the model, we represented the input dataset $\mathrm{X }= [{{\text{x}}}_{1},{{\text{x}}}_{2},{{\text{x}}}_{3}....{{\text{x}}}_{{\text{n}}}]\in {{\text{R}}}^{{\text{p}}*{\text{n}}}$, and the label set ${\text{Y}}\in {\{\mathrm{1,0}\}}^{{\text{q}}*{\text{n}}}$, where ${\text{q}}$ denotes the label dimension, ${\text{p}}$ denotes the feature dimension, and $n$ represents the number of samples. For such representation, the traditional multi-label learning [25] adopts the binary linear regression model to learn matrix ${W}_{p*q}$, as follows:

1 $${\text{arg min}}\left| {\left| {{\text{Y}} - {\text{W}}^{{\text{T}}} {\text{X}}} \right|} \right|_{{\text{F}}}^{2}$$

However, the model has many shortcomings. When the label dimension is large, its accuracy will be reduced. At the same time, the model ignores the correlation between samples. Aiming at this problem, a new model was constructed in this study. To aid easy understanding, the model formulation is divided into several steps as follows.

### Capturing the correlation between samples

To capture internal relation between samples and improve the classification effect of the traditional multi-label model in asthma prediction, we considered using domain information from the sample to enhance robustness. To arrive at our model, a projection matrix $P$ was obtained first to reduce the dimensionality of the feature space and preserve the most discriminative features so that similar sample nodes are closer to each other and their corresponding label nodes are also close to each other. Simultaneously, an affinity graph $W$ was learned on the resulting subspace to capture the domain information. The specific formula is as follows:

2 $$\mathop \sum \limits_{i,j} \left| {\left| {p^{T} x_{i} - p^{T} x_{j} } \right|} \right|_{F}^{2} W_{ij} \Leftrightarrow 2tr\left( {P^{T} XLX^{T} P} \right)$$

where the projection matrix is obtained, such that ${P}^{T}X\to Y$. In order to avoid trivial solutions, we imposed nonnegative and normalized constraints on the graph. Therefore, the above model was transformed into:

3 $$\mathop \sum \limits_{i,j} \left| {\left| {p^{T} x_{i} - p^{T} x_{j} } \right|} \right|_{F}^{2} W_{ij} + \left| {\left| W \right|} \right|_{F}^{2} \quad \quad s.t. 0 \le W_{i,j} \le 1,\mathop \sum \limits_{j} W_{i,j} = 1$$

Specifically, by introducing the affinity matrix $W$, we can further learn the relationship between samples. The value of the $W$ matrix represents the degree of correlation between the similar sample and samples from other classes. That is, the closer the distance between sample nodes, the greater the correlation.

### Affinity graph enhanced classifier

As depicted by Eq. (3), $P$ projects the original feature space into the low-dimensional space to reduce the number of digits in the feature space. The affinity graph is learned on the low-rank subspace to capture the correlation between samples. On this basis, a new classifier $Z$ was constructed to benefit from the domain information through the affinity graph. This strategy helps uncover complex data patterns that hold clinical relevance in the context of asthma. In addition, in order to avoid redundant information in the feature space and make the low-dimensional mapping of data retain the main information in the original data, we introduced an orthogonal constraint ${P}^{T}X{X}^{T}P = I$, and the new optimization model became:

4 $${\text{min }}\left| {\left| {{\text{Y}} - {\text{ZW}}} \right|} \right|_{{\text{F}}}^{2} +\uplambda _{2} \left| {\left| {\text{Z}} \right|} \right|_{{\text{F}}}^{2} \quad \quad {\text{s}}.{\text{t}}.P^{T} XX^{T} P = I$$

**Fig. 1:**
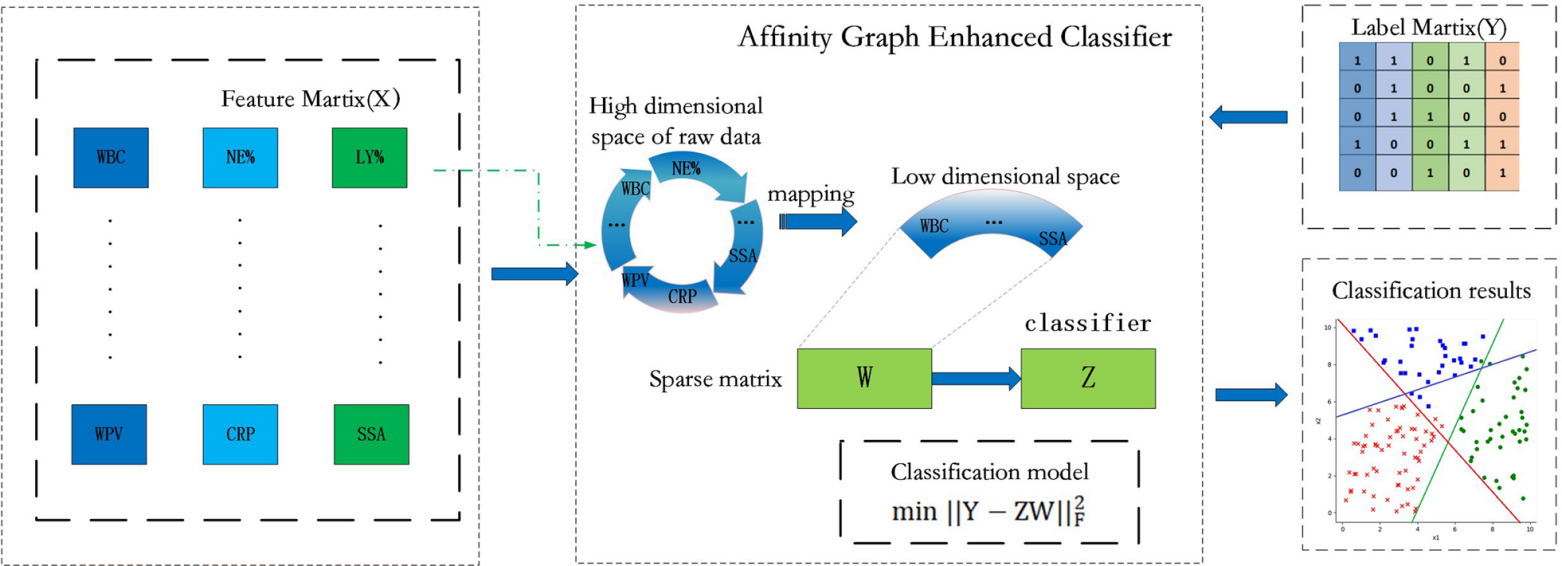
Framework of the proposed method. As can be seen in the figure, the original data is mapped first into a low-dimensional space. A classifier is then constructed to leverage the domain information from the affinity graph for asthma prediction

Furthermore, we introduced an auxiliary variable $M$ through the constraint $W=M$ to make Eq. (4) easier to solve, similar to the previous works [26, 27]. Therefore, combining Eqs. (3) and (4), our objective function was obtained as:

5 $$\begin{aligned} & {\text{min }}\left| {\left| {{\text{Y}} - {\text{ZM}}} \right|} \right|_{{\text{F}}}^{2} +\uplambda _{1} \mathop \sum \limits_{i,j} \left| {\left| {p^{T} x_{i} - p^{T} x_{j} } \right|} \right|_{F}^{2} W_{ij} +\uplambda _{2} \left| {\left| {\text{Z}} \right|} \right|_{{\text{F}}}^{2} +\uplambda _{3} \left| {\left| W \right|} \right|_{F}^{2} \\ & \quad \quad \quad s.t. 0 \le W_{i,j} \le 1,\mathop \sum \limits_{j} W_{i,j} = 1, W = M,P^{T} XX^{T} P = I \\ \end{aligned}$$

where, ${\uplambda }_{1}$, ${\uplambda }_{2}$ and ${\uplambda }_{3}$ denote the regularization parameters used to constrain the second, third, and fourth terms. Figure 1 describes the framework of the proposed method.

### Model optimization

In order to solve our objective function, an efficient optimization algorithm was implemented based on the Augmented LaGrange Multiplier (ALM) strategy [28]. Before that, we obtained the Augmented LaGrange function as follows.

6 $${\text{min }}\left| {\left| {{\text{Y}} - {\text{ZM}}} \right|} \right|_{{\text{F}}}^{2} +\uplambda _{1} \mathop \sum \limits_{i,j} \left| {\left| {p^{T} x_{i} - p^{T} x_{j} } \right|} \right|_{F}^{2} W_{ij} +\uplambda _{2} \left| {\left| {\text{Z}} \right|} \right|_{{\text{F}}}^{2} +\uplambda _{3} \left| {\left| W \right|} \right|_{F}^{2} + tr\left( {Y_{1}^{T} \left( {W - M} \right)} \right) + \frac{{\mu_{1} }}{2}\left| {\left| {W - M} \right|} \right|_{F}^{2}$$

where ${Y}_{1}$ is the LaGrange multiplier, which is necessary for solving constrained problems. Thus, separating the unconnected terms in Eq. (6), the minimization problem and the ideal solution for each variable are given below in no particular order.

#### Z subproblem

Considering only the terms containing Z, we obtained the following optimization function.

7 $${\text{min }}\left| {\left| {{\text{Y}} - {\text{ZM}}} \right|} \right|_{{\text{F}}}^{2} +\uplambda _{2} \left| {\left| {\text{Z}} \right|} \right|_{{\text{F}}}^{2}$$

Thus, expanding the first item in Eq. (7), we arrived at:

8 $$\left| {\left| {{\text{Y}} - {\text{ZM}}} \right|} \right|_{{\text{F}}}^{2} = {\text{tr}}\left( {\left( {{\text{Y}} - {\text{ZM}}} \right)^{{\text{T}}} \left( {{\text{Y}} - {\text{ZM}}} \right)} \right) = {\text{tr}}\left( {{\text{Y}}^{{\text{T}}} {\text{Y}} - {\text{Y}}^{{\text{T}}} {\text{ZM}} - {\text{M}}^{{\text{T}}} {\text{Z}}^{{\text{T}}} {\text{Y}} - {\text{M}}^{{\text{T}}} {\text{Z}}^{{\text{T}}} {\text{ZM}}} \right)$$

After considering only variable Z, we obtained:

9 $${\text{min tr}}\left( {{\text{M}}^{{\text{T}}} {\text{Z}}^{{\text{T}}} {\text{ZM}} - 2{\text{M}}^{{\text{T}}} {\text{Z}}^{{\text{T}}} {\text{Y}}} \right) +\uplambda _{2} {\text{tr}}\left( {{\text{Z}}^{{\text{T}}} {\text{Z}}} \right)$$

Consequently, a partial derivative of $Z$ yielded:

10 $$\partial_{Z} = \left( {{\text{MM}}^{{\text{T}}} {\text{Z}}^{{\text{T}}} - MY^{T} +\uplambda _{2} {\text{Z}}^{{\text{T}}} } \right)^{T} =\uplambda _{2} {\text{Z}} + {\text{ZMM}}^{{\text{T}}} - {\text{YM}}^{{\text{T}}}$$

Setting the Eq. (10) equal to 0, that is, ${\uplambda }_{2}{\text{Z}}+{{\text{ZMM}}}^{{\text{T}}}-{{\text{YM}}}^{{\text{T}}}=0$, the optimal solution of $Z$ was obtained through the following formula:

11 $${\text{Z}} = {\text{YM}}^{{\text{T}}} \left( {\uplambda _{2} {\text{I}} + {\text{MM}}^{{\text{T}}} } \right)^{ - 1}$$

#### P subproblem

12 $${\text{min }}\uplambda _{1} \mathop \sum \limits_{i,j} \left| {\left| {p^{T} x_{i} - p^{T} x_{j} } \right|} \right|_{F}^{2} W_{ij} \quad {\text{s}}.{\text{t}}.{\text{P}}^{{\text{T}}} {\text{XX}}^{{\text{T}}} {\text{P }} = {\text{ I}}$$

Expanding the above optimization function, Eq. (12) can be rewritten as:

13 $${\text{min }}\uplambda _{1} {\text{tr}}\left( {{\text{P}}^{{\text{T}}} {\text{XL}}_{{\text{b}}} {\text{X}}^{{\text{T}}} {\text{P}}} \right) \quad {\text{s}}.{\text{t}}.{\text{P}}^{{\text{T}}} {\text{XX}}^{{\text{T}}} {\text{P }} = {\text{ I}}$$

Therefore, using Lagrange multiplier method, we obtained:

14 $${\text{min }}\uplambda _{1} {\text{tr}}\left( {{\text{P}}^{{\text{T}}} {\text{XL}}_{{\text{b}}} {\text{X}}^{{\text{T}}} {\text{P}}} \right) - \left( {{\text{P}}^{{\text{T}}} {\text{XX}}^{{\text{T}}} {\text{P}} - {\text{I}}} \right)$$

A partial derivative of $P$ yielded:

15 $$\partial_{P} = \left( {\uplambda _{1} {\text{P}}^{{\text{T}}} {\text{XL}}_{{\text{b}}} {\text{X}}^{{\text{T}}} - \left( {{\text{P}}^{{\text{T}}} {\text{XX}}^{{\text{T}}} } \right)} \right)^{T}$$

Setting Eq. (15) equal to 0,

16 $$\uplambda _{1} P = \left( {{\text{XL}}_{{\text{b}}} {\text{X}}^{{\text{T}}} } \right)^{ - 1} {\text{XX}}^{{\text{T}}} {\text{P}}$$

Finally, the optimal value of matrix $P$ was obtained by finding the eigenvector corresponding to matrix ${({{\text{XL}}}_{{\text{b}}}{{\text{X}}}^{{\text{T}}})}^{-1}{{\text{XX}}}^{{\text{T}}}$.

#### M subproblem

17 $${\text{min }}\left| {\left| {{\text{Y}} - {\text{ZM}}} \right|} \right|_{{\text{F}}}^{2} + tr\left( {Y_{1}^{T} \left( {W - M} \right)} \right) + \frac{{\mu_{1} }}{2}\left| {\left| {W - M} \right|} \right|_{F}^{2}$$

As mentioned previously, ${Y}_{1}$ is the Lagrange multiplier, and ${\upmu }_{1}>0$ is the penalty parameter. Equation (17) can be rewritten as:

18 $$\begin{aligned} & {\text{min tr}}\left( {\left( {{\text{Y}} - {\text{ZM}}} \right)^{{\text{T}}} \left( {{\text{Y}} - {\text{ZM}}} \right)} \right) + tr\left( {Y_{1}^{T} \left( {W - M} \right)} \right) + \frac{{\mu_{1} }}{2}\left| {\left| {W - M} \right|} \right|_{F}^{2} \\ & \quad = {\text{min tr}}\left( {{\text{Y}}^{{\text{T}}} {\text{Y}} - {\text{Y}}^{{\text{T}}} {\text{ZM}} - {\text{M}}^{{\text{T}}} {\text{Z}}^{{\text{T}}} {\text{Y}} - {\text{M}}^{{\text{T}}} {\text{Z}}^{{\text{T}}} {\text{ZM}}} \right) + tr\left( {Y_{1}^{T} \left( {W - M} \right)} \right) \\ & \quad \quad + \frac{{\mu_{1} }}{2}\left( {\left( {W - M} \right)^{T} \left( {W - M} \right)} \right) \\ \end{aligned}$$

Extracting only variables related to $M$:

19 $${\text{min tr}}\left( {{\text{M}}^{{\text{T}}} {\text{Z}}^{{\text{T}}} {\text{ZM}} - 2{\text{M}}^{{\text{T}}} {\text{Z}}^{{\text{T}}} {\text{Y}}} \right) + tr\left( {Y_{1}^{T} M} \right) + \frac{{\mu_{1} }}{2}\left( {{\text{M}}^{{\text{T}}} {\text{M}} - 2{\text{W}}^{{\text{T}}} M} \right)$$

As with the other variables, a partial derivative of $M$ yielded:

20 $$\partial_{M} = \left( {2{\text{M}}^{{\text{T}}} {\text{Z}}^{{\text{T}}} {\text{Z}} - 2{\text{Y}}^{{\text{T}}} Z - Y_{1}^{T} + \mu_{1} {\text{M}}^{{\text{T}}} - \mu_{1} {\text{W}}^{{\text{T}}} } \right)^{T}$$

Setting Eq. (20) = 0, the optimal solution of $M$ was obtained through the following formula:

21 $${\text{M}} = \left( {\frac{2}{{\mu_{1} }}{\text{Z}}^{{\text{T}}} {\text{Z}} + {\text{I}}} \right)^{ - 1} \left( {{\text{W}} + \frac{1}{{\mu_{1} }}Y_{1} + \frac{2}{{\mu_{1} }}{\text{Z}}^{{\text{T}}} Y} \right)$$

#### W subproblem

22 $$\mathop \sum \limits_{i,j} \left| {\left| {p^{T} x_{i} - p^{T} x_{j} } \right|} \right|_{F}^{2} W_{ij} +\uplambda _{3} \left| {\left| W \right|} \right|_{F}^{2} + tr\left( {Y_{1}^{T} \left( {W - M} \right)} \right) + \frac{{\mu_{1} }}{2}\left| {\left| {W - M} \right|} \right|_{F}^{2}$$

Expanding Eq. (22), we arrived at:

23 $$\mathop \sum \limits_{i,j} \left| {\left| {p^{T} x_{i} - p^{T} x_{j} } \right|} \right|_{F}^{2} W_{ij} + \frac{{\mu_{1} }}{2}\left\| {W - M + \frac{{Y_{1} }}{{\mu_{1} }}} \right\|^{2} +\uplambda _{3} \left| {\left| W \right|} \right|_{F}^{2}$$

By making ${\text{M}}-\frac{{{\text{Y}}}_{1}}{{\upmu }_{1}}={\text{U}}$, Eq. (23) can be rewritten as:

24 $$\begin{aligned} & min_{W} \mathop \sum \limits_{i,j} \left| {\left| {p^{T} x_{i} - p^{T} x_{j} } \right|} \right|_{F}^{2} W_{ij} + \frac{{\mu_{1} }}{2}\left\| {W - U} \right\|^{2} +\uplambda _{3} \left| {\left| W \right|} \right|_{F}^{2} \\ & \quad \quad \quad \quad s.t. 0 \le W_{i,j} \le 1,\mathop \sum \limits_{j} W_{i,j} = 1 \\ \end{aligned}$$

Because Eq. (24) is independent for each $i$, we solved ${{\text{W}}}_{{\text{i}}}$ separately as follows:

25 $$\begin{aligned} & min_{{W_{i} }} \mathop \sum \limits_{i,j} \left| {\left| {P^{T} X_{i} - P^{T} X_{i} } \right|} \right|^{2} W_{i,j} + \frac{{\mu_{1} }}{2}\left\| {W_{i} - U_{i} } \right\|^{2} +\uplambda _{3} |\left| {W_{i} } \right||^{2} \\ & \quad \quad \quad \quad s.t. 0 \le W_{i,j} \le 1,\mathop \sum \limits_{j} W_{i,j} = 1 \\ \end{aligned}$$

Denoting ${e}_{i,j}=\left|\left|{P}^{T}{X}_{i}-{P}^{T}{X}_{i}\right|\right|,{w}_{v}=\frac{{\mu }_{1}}{2}$, we rewrite Eq. (25) in the following way.

26 $$\begin{aligned} & min_{{W_{i} }} \frac{1}{2}\left\| {W_{i} + \frac{{e_{i} }}{{2\uplambda _{3} }}} \right\|^{2} + \frac{1}{{2\uplambda _{3} }}\left\| {U_{i} - W_{i} } \right\|^{2} \\ & \quad \quad s.t. 0 \le W_{i,j} \le 1,\mathop \sum \limits_{j} W_{i,j} = 1 \\ \end{aligned}$$

27 $$\begin{aligned} L\left( {W_{i} ,\eta ,\xi ,} \right) & = \frac{1}{2} \left| {\left| {W_{i} + \frac{{e_{i} }}{{2\uplambda _{3} }}} \right||^{2} + \frac{1}{{2\uplambda _{3} }}} \right|\left| {U_{i} - W_{i} } \right||^{2} \\ & \quad - \eta \left( {1^{T} W_{i} - 1} \right) - \xi^{T} W_{i} \\ \end{aligned}$$

*η* is the scalar of the Lagrange coefficient, and *ξ* is the vector of the Lagrange coefficient. Taking a partial derivative of ${{\text{W}}}_{{\text{i}}}$, we obtained:

28 $$W_{i} + \frac{{e_{i} }}{{2\uplambda _{3} }} - \frac{1}{{\uplambda _{3} }} w_{v} \left( {U_{i} - W_{i} } \right) - \eta 1 - \xi = 0$$

The jth term of ${{\text{W}}}_{{\text{i}}}$ in the equation is:

29 $$W_{i,j} + \frac{{e_{i,j} }}{{2\uplambda _{3} }} - \frac{1}{{\uplambda _{3} }} w_{v} \left( {U_{i,j} - W_{i,j} } \right) - \eta - \xi_{j} = 0$$

By following the KKT conditions [29], we obtained ${W}_{i,j}$ through the following formula.

30 $$W_{i,j} = \left( {\frac{{ - \frac{{e_{i,j} }}{2} + w_{v} U_{i,j} + {\uplambda }_{4} \eta }}{{{\uplambda }_{3} + w_{v} }}} \right) +$$

Furthermore, $$- \frac{{e_{i,k} }}{2} + w_{v} U_{i,k} +\uplambda _{3} \eta > 0\;{\text{and}}\; - \frac{{e_{i,k + 1} }}{2} + w_{v} U_{i,k + 1} +\uplambda _{3} \eta \le 0,\eta = \frac{1}{k} \left( {1 + \frac{{ w_{v} }}{{\uplambda _{3} }} + \mathop \sum \limits_{h = 1}^{k} \frac{{e_{i,h} }}{{2\uplambda _{3} }}} \right),$$

$$\left\{ \begin{aligned} & \lambda_{4} > \frac{{ke_{i,k} - \mathop \sum \nolimits_{h = 1}^{k} e_{i,h} - 2k w_{v} U_{i,k} - 2 w_{v} }}{2} \hfill \\ & \lambda_{4} \le \frac{{ke_{i,k + 1} - \mathop \sum \nolimits_{h = 1}^{k} e_{i,h} - 2k w_{v} U_{i,k + 1} - 2 w_{v} }}{2} \hfill \\ \end{aligned} \right.$$

$$\uplambda _{3} = \frac{{ke_{i,k + 1} - \mathop \sum \nolimits_{h = 1}^{k} e_{i,h} - 2k w_{v} U_{i,k + 1} - 2 w_{v} }}{2}.$$

31 $$W_{i,j} = \left\{ {\begin{array}{*{20}l} {\frac{{e_{i,k + 1} - e_{i,j} + 2 w_{v} U_{i,j} - 2 w_{v} U_{i,k + 1} }}{{ke_{i,k + 1} - \mathop \sum \nolimits_{h = 1}^{k} e_{i,h} - 2k w_{v} U_{i,k + 1} + 2\mathop \sum \nolimits_{h = 1}^{k} w_{v} U_{i,h} }},} & {j \le k} \\ {0,} & {{\text{j}} > {\text{k}}} \\ \end{array} } \right.$$

For the detailed derivation and proof of Eq. (31), refer to reference [30]. A summary of the complete solution of our proposed model is captured in Algorithm1.

### Compared classification algorithms

Five classification algorithms were used to build classification models for comparison with our AGEC model. They are, multi-label learning with feature-induced labeling information enrichment (MLFE) [31], support vector machines (SVM), exclusivity regularized machine (ERM) [32], support vector regression (SVR) [33], and multi-class fuzzily weighted AdaBoost (FWAdaBoost) [34]. We considered these algorithms for comparison because they use a similar strategy to AGEC or because they are often used for building asthma predictive models. For example, MLFE is a multi-label learning algorithm like ours. SVM and SVR are commonly used for building asthma predictive models due to their excellent generalization ability [35]. ERM and FWAdaBoost, which is based on AdaBoost [36] uses the ensemble learning strategy, which is well-known to improve the performance of single-task learning models.

**Algorithm 1:**
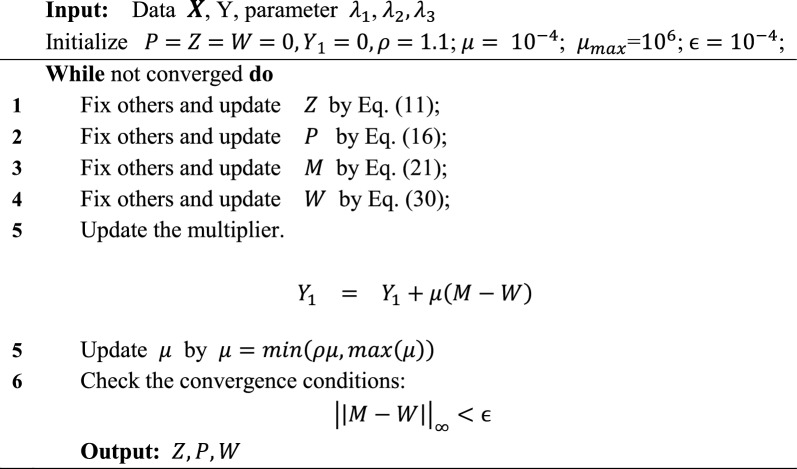
The Algorithm of the proposed model

### Evaluation

The experimental results were captured in terms of accuracy (ACC) and the area under the receiver operating characteristic (ROC) curve (AUC). These metrics were utilized to characterize and compare the performance of the various classification algorithms in asthma prediction. While ACC measures how well a model can correctly predict class labels of the instances in the test set, AUC measures the overall performance of a classifier by evaluating its ability to distinguish between positive and negative instances. Unlike ACC, AUC is insensitive to changes in class distribution.

## Results

### Experiment settings

The comparison algorithms and our AGEC algorithm were implemented using MATLAB R2016a installed on a Windows 10 computer system. In order to reasonably evaluate the effectiveness of our model, two sets of experiments were performed. The first set examined the performance of each algorithm using all 24 clinical indicators. The second investigated the effect of different subsets of the features on the performance of the proposed method. In each experiment, we first divided the dataset into a training set and a held-out testing set with a ratio of 1:1. Then twofold cross-validation was performed on the training dataset for parameter tuning. We selected 2 based on the relatively small size of our dataset. Moreover, the grid search strategy was also applied to tune the hyperparameters during cross-validation. The optimal hyperparameters for our best AGEC were ${\uplambda }_{1}=8*{10}^{-4}$, ${\uplambda }_{2}=2*{10}^{-5}$, ${\uplambda }_{3}=1.8*{10}^{-4}$.

### Evaluation of the prediction models

**Fig. 2:**
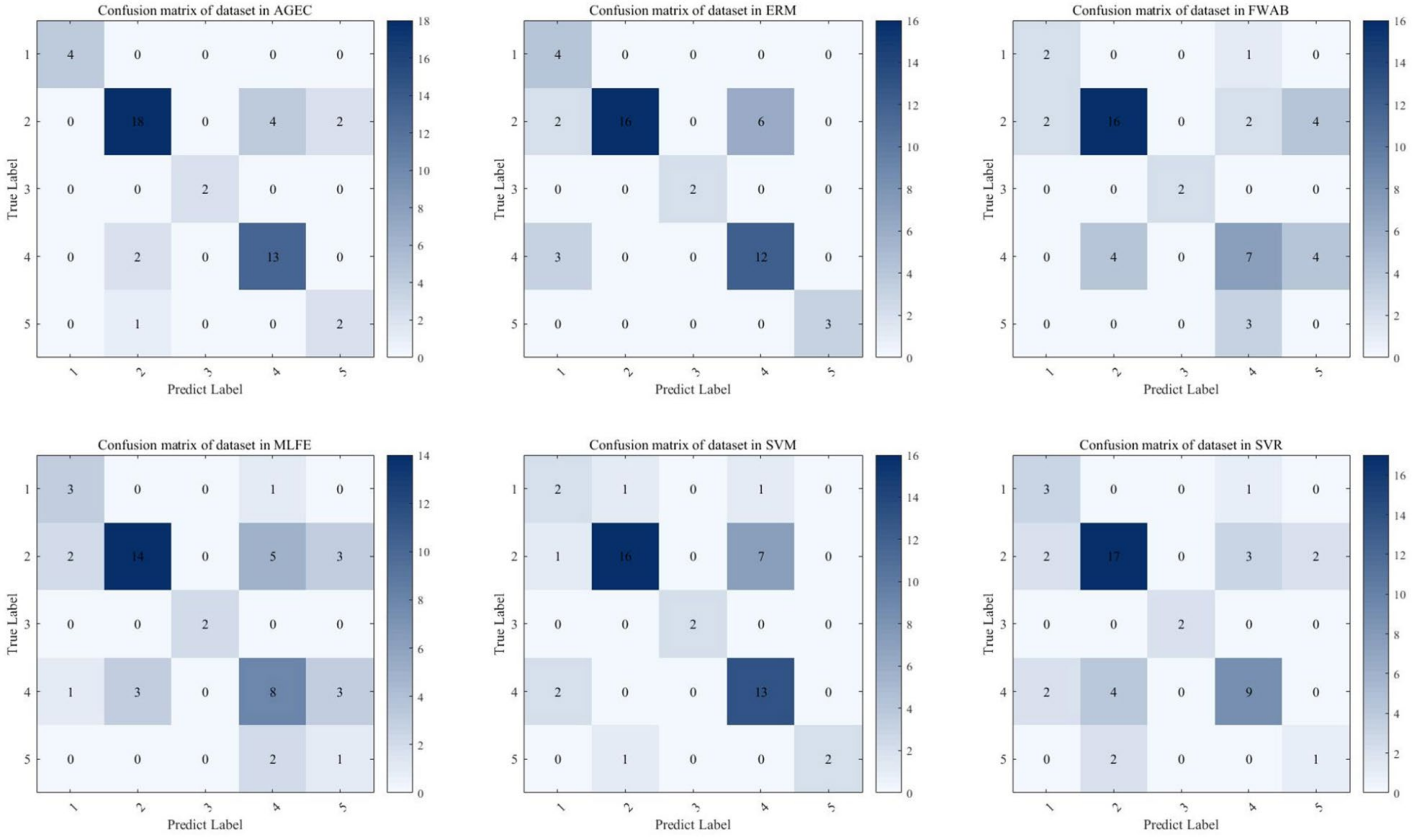
The confusion matrix obtained for each of the six approaches

Table 3 displays the performance in terms of the accuracy of various models, including AGEC, in asthma prediction. As can be seen from the results, AGEC obtained an accuracy of 72.50%, which is significantly higher than other models. Although there is a seemingly insignificant gap of 2.7% between AGEC and the SVM model, the gap widens in terms of AUC, as shown in Table 4. Specifically, AGEC obtained an AUC of 74.01%, which is significantly higher than SVM by over 3% and much higher than the other models. This suggests that our model has the better capability in distinguishing between asthmatic and non-asthmatic patients. Moreover, the $p$ value also suggest that the differences between our model and other models are statistically significant, and the effect of our model does not exist by chance. In addition, to more specifically demonstrate the advantages of our proposed model, Fig. 2 shows the confusion matrix obtained for each of the six models. As can be seen in the figure, the shadow on the diagonal of our AGEC is deeper than that on other models, which means that our model can make more correct classification results than other models. Meanwhile, the shadow on the non-diagonal is less than that on other models, which means that our model can predict fewer wrong results.

**Table 3:** ACC of AGEC compared with different models

	MLFE	SVM	ERM	SVR	FWAdaBoost	AGEC
ACC	0.6098	0.6980	0.6840	0.6401	0.6102	**0.7250**

The value in bold font symbolizes the best performance

**Table 4:** AUC and P values of AGEC compared with different models

Algorithm	Prediction accuracy (area under the curve)	
	AUC	*P* value
MLFE	0.5201 ± 0.016	0.0304
SVM	0.7034 ± 0.012	0.0302
ERM	0.6632 ± 0.030	0.0298
SVR	0.6312 ± 0.022	0.0305
FWAdaBoost	0.7014 ± 0.002	0.0301
AGEC	**0.7401 ± 0.021**	0.0305

The value in bold font symbolizes the best performance

Additionally, we also conducted comparison with some regression models: Logistic Regression, Random Forest (RF) and Lasso. The results, as presented in Table 5, indicate that the accuracy of Logistic Regression (59.24%), RF (54.21%), and Lasso (56.01%) is notably lower than the accuracy achieved by the previously compared methods. This comparison highlights the superior performance of our proposed method in the context of asthma prediction. Moreover, the observed lower accuracy of Logistic Regression, RF, and Lasso can be attributed to several factors. Logistic Regression may struggle to capture the complex non-linear relationships present in the data, leading to suboptimal predictive performance. RF, while robust in certain contexts, may face challenges in handling the specific characteristics of the asthma prediction task. Lasso, being a feature selection method, may not effectively discern the important features contributing to asthma prediction, resulting in reduced accuracy.

**Table 5:** ACC of AGEC compared with different regression models

	Logistic regression	Random forest	Lasso	AGEC
ACC	0.5924	0.5421	0.5601	**0.7250**

The value in bold font symbolizes the best performance

### Impact of different subsets of features on the effectiveness of AGEC

**Fig. 3:**
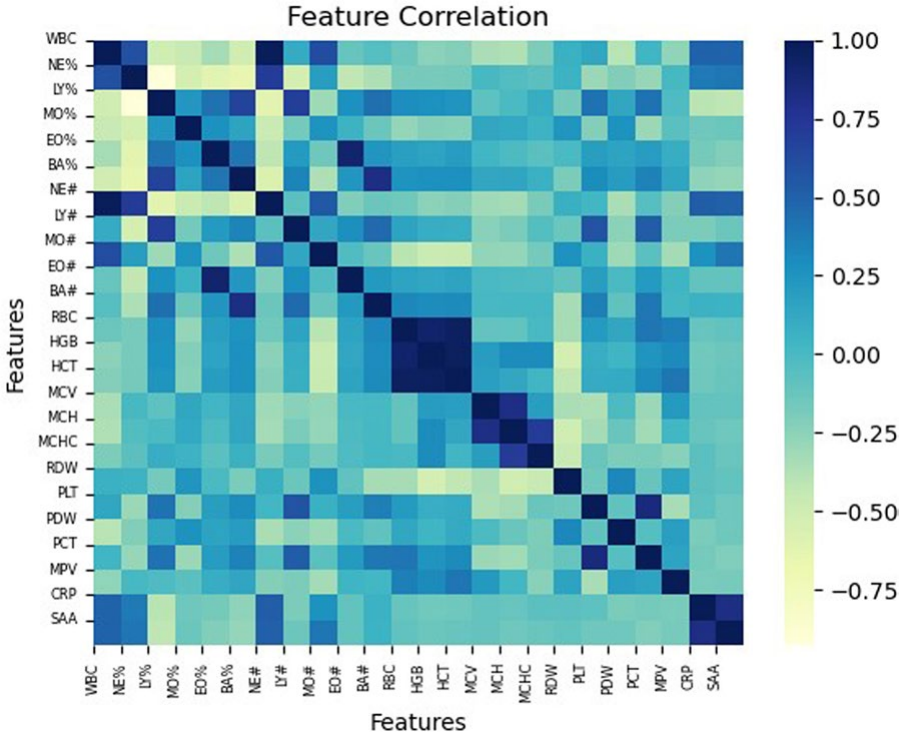
A heatmap visualization of the correlation between features

This experiment aimed to determine the discriminability of various feature sets in asthma prediction. Here, we explored three groups of features. The first set of features was extracted by considering prior knowledge from relevant medical literature, such as [37, 38], yielding a group consisting of 14 key features. The characteristics of these features are described as follows: WBC, LY%, MO%, LY#, MO#, EO#, BA#, RBC, MCH, MCHC, RDW, PLT, PDW, MPV. Based on this, we further investigated the correlation between features using a heat map. As may be noticed in Fig. 3, we observed that PDW and MPV among the indicators of blood routine have a great impact on the final results, so we take these two indicators as the center. Then, the heat map was used to find the features that are highly correlated with those two indicators, leading to two additional sets of features. Thus, the second group has 13 features: PDW, MPV, RDW, BA%, EO%, MO%, LY#, PCT, PLT, MCV, HCT, HGB, and RBC. The third group has 15 features, which are shown as follows. MPV, PCT, PDW, RDW, MCH, MCV, HCT, HGB, RBC, BA#, EO#, BA%, EO%, LY%, and NE%.

**Fig. 4:**
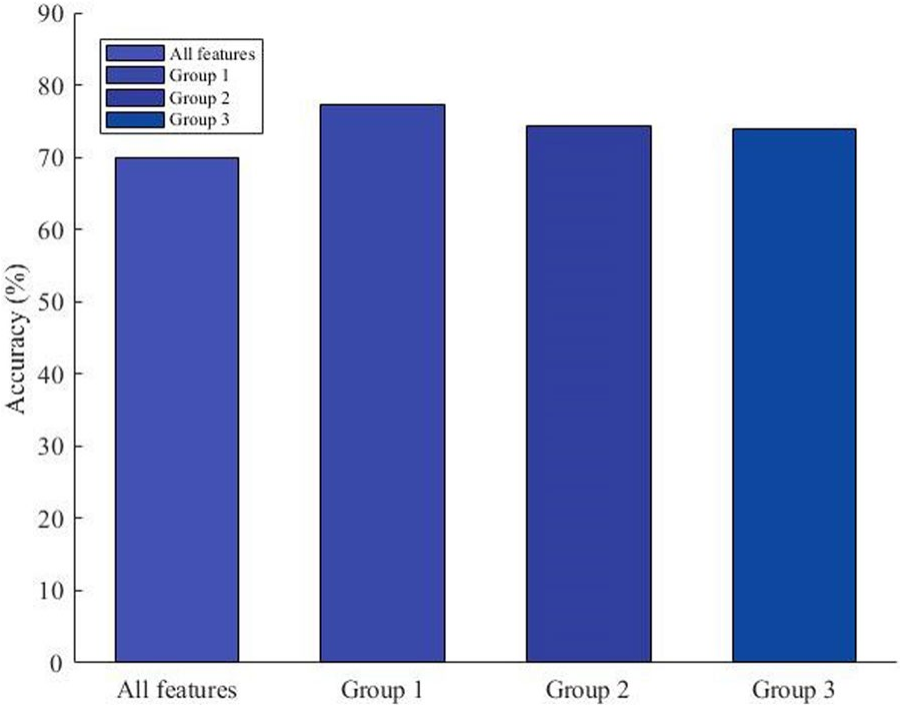
The ACC of AGEC on different set of features

According to the results in Fig. 4, our model obtained an accuracy of 78.18%, 75.29% and 72.92% under the first, second and third groups, respectively. Notably, AGEC demonstrated the highest accuracy (78.18%) in the first set, indicating that the selected features were particularly effective in distinguishing between groups. In contrast, the model achieved slightly lower accuracies of 75.29% and 72.92%, respectively, for the second and third sets, suggesting that some of the features employed in these sets were not as discriminatory. Interestingly, it can be observed that the third group, despite having more features (15), did not outperform the second group (13 features), meaning that the additional features may not have significantly contributed to the classification task. These findings thus underscore the fact that not all added features would necessarily improve the performance of a classification model.

**Fig. 5:**
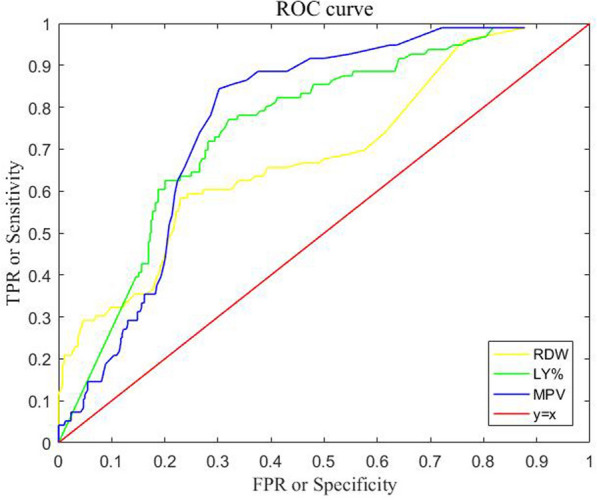
The ROC curve of the true positive rate against the false positive rate with respect to MPV, LY% and RDW indicators

As a result of the above, we further conducted experiments on each of the 24 features to determine which input features are most salient. Based on these experiments, we present a graphical representation of the performance of the classification model using a ROC curve. This plots the true positive rate (TPR), also known as sensitivity, against the false positive rate (FPR), also referred to as specificity. As shown in Fig. 5, we only display results of MPV, LY% and RDW with more obvious effects. Accordingly, it can be observed that the curve area formed by these three indicators is greater than $y=x$, meaning that our model has practical significance in the three indicators. At the same time, it can also be observed that MPV has a better effect on the classification of asthma compared to other indicators.

## Discussion

In this study, we presented a novel model for asthma prediction that incorporates an affinity graph enhanced classifier and utilized previously unexplored clinical indicators. This combination sets our study apart from previous works, offering distinct advantages and contributing to the field of medical predictive modeling.

One of the key advantages of our approach was the integration of affinity graph to capture correlations between samples. This aspect of our approach enhanced the ability of our model to capture intricate interactions within the data and improve overall prediction performance. In addition to the use the affinity graph, our study focused on utilizing unique clinical predictors for asthma prediction. We extracted 24 clinical indicators, including blood count differentials and red blood cell indices. As far as we know, the selected predictors have not been previously utilized “solely” for the training of ML models in the context of asthma prediction. This inclusion thus expands the scope of predictors used in asthma prediction models and can potentially uncover new insights into the disease. Moreover, our study demonstrated that utilizing these unique clinical predictors alone can achieve competitive performance, with an ACC of 72.50% and an AUC of 74.01%, as shown in Tables 3 and 4, respectively. This highlights the effectiveness of our proposed model, showing that the employed clinical indicators can provide meaningful and discriminative information for asthma prediction. Furthermore, the use of these clinical predictors offers advantages in terms of simplicity, interpretability and generalization. For example, collecting and integrating various data sources can be challenging and time-consuming, whereas our approach simplifies the prediction process by focusing exclusively on clinical data, which are often readily available in medical settings. This streamlined approach enhanced the ease of implementation, and, we hope that the clinical community may cautiously consider the adoption of our model to facilitate prompt detection and management of asthma to avoid exacerbations and hospitalizations.

In addition to the improvements offered from the above two perspective, it is noteworthy to highlight the robustness of our approach. While previous studies, such as [9, 39], have often relied on traditional ML algorithms and utilized data from multiple sources, such as age, gender, lung function measurements, and medical history to make predictions, our study demonstrated that using a focused set of unique clinical predictors can achieve a comparable or even superior performance if the predictive model can capture the correlation between samples. To further emphasize this, we examined the results reported in existing literature for asthma prediction models. While the specific studies may vary, a comprehensive review of recent works [40] revealed that the performance accuracy of most asthma predictive models is generally > 65%. In comparison, our study achieved an accuracy of 70% using only the selected clinical predictors.

Furthermore, based on the evaluation of the effect of three different subsets of features on the performance of AGEC, we found that the accuracy of the proposed model can reach 78.18%, with the accuracy across all three sets ranging from 72.92% to 78.18%. This variation underpins the importance of feature selection in enhancing the performance of classification models. More specifically and consistent with previous medical studies by Panet al. [37] and Zhu et al. [38], the first group, with its specific set of features, demonstrated the highest accuracy. This suggests that the co-existence of certain indicators, such as WBC, LY%, MO%, LY#, MO#, EO#, BA#, RBC, MCH, MCHC, RDW, PLT, PDW, and MPV, can play a crucial role in distinguishing asthma cases. Besides, the observed significance of MPV in our study suggests that platelet-related factors may play a role in diagnosing asthma. This finding aligns with emerging evidence in [41–43] that implicates platelet activation and inflammation in the pathogenesis of asthma. Additionally, the differential impact of LY% and RDW on asthma classification underscores the intricate interplay between lymphocyte percentages and red cell distribution width in the context of asthma-related processes. These insights provide a foundation for exploring potential biomarkers related to immune response and erythropoiesis in asthma. Therefore, it is also hoped that this knowledge will further guide clinicians in prioritizing these indicators for prompt and accurate diagnosis of asthma, ultimately reducing the burden on healthcare systems. Another advantage of our approach is its potential for easy extension to other diseases detection. This flexibility demonstrates the broader applicability and impact of our study. Nonetheless, even though the proposed approach has been validated to be effective, our study may have been limited by the size of the dataset. Although we tried to mitigate such effects via the incorporation of dimensionality reduction in our model, we believe that, in the future, the accuracy of AGEC can be further improved by increasing the sample population. Moreover, recent studies such as [44], have found that the level of heavy metals in serum was higher in individuals with acute exacerbation of Chronic Obstructive Lung Disease (COPD); therefore, in future work, we hope to employ a combination of these features with the other blood markers used in this study to enhance accuracy.

## Conclusions

In this paper, we proposed a new method for predicting asthma using an affinity graph enhanced classifier. Our approach specifically addressed the limitation of existing models in terms of capturing the correlation between data samples. As a result, the accuracy of our model was improved in asthma prediction. This was accomplished by utilizing domain knowledge through the affinity graph. Compared with existing state-of-the-art related models concerning ACC and AUC, our AGEC demonstrated significant improvement in asthma prediction. To the best of our knowledge, this is the first study that directly exploits the affinity graph for classification tasks, and the results have shown its effectiveness. In addition, the proposed approach is completely data-driven and can easily be generalized to other prediction tasks, thus providing a framework for future research. Moreover, beyond the immediate scope of asthma prediction, the implications of our findings extend to the broader context of asthma management and healthcare. The enhanced accuracy and novel methodology introduced by AGEC holds potential benefits for improving early asthma detection, thus enabling more proactive and targeted interventions. This, in turn, could contribute to the optimization of patient care, reduction of healthcare costs, and the overall enhancement of asthma management strategies.

## Data Availability

The authors declare that all data supporting the findings of this study are available withing the article and its additional files or by contacting the corresponding author upon reasonable request.
